# Novel nomogram to predict the overall survival of postoperative patients with gastric signet

**DOI:** 10.1186/s12876-023-02915-z

**Published:** 2023-08-16

**Authors:** Donghui Liu, Ran Ding, Liru Wang, Enhong Shi, Xiaoxue Li, Chenyao Zhang, Yan Zhang, Xuyao Wang

**Affiliations:** 1 School of Life Science and Technology, Harbin Institute of Technology, Harbin, China; 2 Department of Oncology, Heilongjiang Provincial Hospital, Harbin, China; 3 Department of Pharmacy, Harbin Second Hospital, Harbin, China

**Keywords:** Nomogram, Signet ring cell carcinoma, Risk factors, Gastric cancer, Prognosis

## Abstract

**Background:**

The TNM staging system cannot accurately predict the prognosis of postoperative gastric signet ring cell carcinoma (GSRC) given its unique biological behavior, epidemiological features, and various prognostic factors. Therefore, a reliable postoperative prognostic evaluation system for GSRC is required. This study aimed to establish a nomogram to predict the overall survival (OS) rate of postoperative patients with GSRC and validate it in the real world.

**Methods:**

Clinical data of postoperative patients with GSRC from 2002 to 2014 were collected from the Surveillance, Epidemiology, and End Results database and randomly assigned to training and internal validation sets at a 7:3 ratio. The external validation set used data from 124 postoperative patients with GSRC who were admitted to the Affiliated Tumor Hospital of Harbin Medical University between 2002 and 2014. The independent risk factors affecting OS were screened using univariate and multivariate analyses to construct a nomogram. The performance of the model was evaluated using the C-index, receiver operating characteristic curve (ROC), calibration curve, decision analysis (DCA) curve, and adjuvant chemotherapy decision analysis.

**Results:**

Univariate/multivariate analysis indicated that age, stage, T, M, regional nodes optimized (RNE), and lymph node metastasis rate (LNMR) were independent risk factors affecting prognosis. The C-indices of the training, internal validation, and external validation sets are 0.741, 0.741, and 0.786, respectively. The ROC curves for the first, third, and fifth years in three sets had higher areas under the curves, (training set, 0.782, 0.864, 0.883; internal validation set, 0.781, 0.863, 0.877; external validation set, 0.819, 0.863, 0.835). The calibration curve showed high consistency between the nomogram-predicted 1-, 3-, and 5-year OS and the actual OS in the three queues. The DCA curve indicated that applying the nomogram enhanced the net clinical benefits. The nomogram effectively distinguished patients in each subgroup into high- and low-risk groups. Adjuvant chemotherapy can significantly improve OS in high-risk group (*P* = 0.034), while the presence or absence of adjuvant chemotherapy in low-risk group has no significant impact on OS (*P* = 0.192).

**Conclusions:**

The nomogram can effectively predict the OS of patients with GSRC and may help doctors make personalized prognostic judgments and clinical treatment decisions.

## Introduction

Gastric cancer (GC) is a prevalent malignancy, with over one million new cases and approximately 783,000 deaths reported annually, according to the World Health Organization’s global cancer statistics for 2018. Its incidence and mortality rates rank fifth and third worldwide, respectively, imposing a significant burden on global health [1]. Although the incidence rate of GC shows a downward trend worldwide, that of gastric signet ring cell carcinoma (GSRC) shows a steady upward trend, particularly in the United States and Europe, where it continues to increase, accounting for 15.9–17% of new cases of adenocarcinoma [2–4]. Although the incidence rate in Asia is relatively low, it increases annually [5]. Therefore, the prevention and treatment of GSRC should be highly valued.

The fourth edition of the 2010 WHO Classification of Digestive System Tumors redefined GSRC as a subtype of low-adhesion gastric cell carcinoma characterized by clear cytoplasmic mucin in the center of the cell, which pushes the nucleus to one side. This subtype accounts for more than 50% of tumors [6]. Mucins are secreted by cells and have potential carcinogenic effects [7]. This classification and definition indicate that the occurrence and development of GSRC involve two unique pathological processes: the loss of intercellular adhesion molecules and the accumulation of mucin in the cytoplasm [8]. In addition, in the early stages of GSRC, tumor cells are widely distributed in the mucosal layer, and the speed of diffusion to the submucosa is relatively slow. However, it metastasizes rapidly once it spreads into the submucosa [9].

The epidemiological features of GSRC differ significantly from those of the other types of GA. GSRC is more common in women than gastric adenocarcinoma, with a male-to-female ratio of approximately 1, while less than half of all sampled women have gastric adenocarcinoma. The affected patients are relatively young, with an average age of approximately 61.9 years, whereas the age of onset of gastric adenocarcinoma is approximately 68.7 years [10]. Related reports indicate that, compared to other histological types of GC, GSRC is more common in women, youth, malnourished patients, and those with larger tumors [11]. Simultaneously, studies have shown that the prognosis of patients with GSRC is associated with advanced age, linitis, and adjacent organ involvement [11]. Additionally, patients with GSRC have a prognosis similar to that of patients with other types of early GC. In patients with advanced GC, prognosis is closely related to age, race, tumor size, AJCC stage, T stage, and postoperative adjuvant therapy [12].

In terms of postoperative adjuvant therapy, the NCCN and CSCO guidelines don’t mention any differences in treatment regimens between GSRC and non-GSRC patients [13, 14]. However, studies have shown that GSRC has lower chemical sensitivity compared to non-GSRC, especially in response to 5FU or platinum drugs, and this regimen is most commonly used for adjuvant chemotherapy during the perioperative period [15]. Taxane based treatment may be more effective for GSRC [16], but this conclusion is also controversial, because Chen et al. found that docetaxel based chemotherapy benefited mixed GSRC when they gave docetaxel or Oxaliplatin chemotherapy in different GSRC tissues. However, in pure GSRC, the results were contradictory, and there was no significant difference between the two kinds of chemotherapy [17].

GSRC has unique biological behavior and epidemiological features, and multiple factors influence its prognosis. However, the current GC TNM staging system cannot accurately predict the prognosis of patients with postoperative GSRC. Therefore, a reliable postoperative prognostic evaluation system for GSRC is urgently required.

This study aimed to construct a GSRC nomogram prognostic evaluation model based on large-sample clinical data from the Surveillance, Epidemiology, and End Results (SEER) database and validate it realistically to assist clinicians in making personalized prognosis judgments and treatment decisions. This manuscript is written following the TRIPOD checklist.

## Methods

### Data acquisition and determination of clinical features

The study utilized the International Classification of Oncological Diseases (ICD-O) and clinical data and follow-up information on postoperative GC from the SEER database (https://seer.cancer.gov/) (SEER_1975_2016) for the period between January 1, 2002, and April 31, 2014. To reduce noise, we eliminated uncertain data, including Ti, Tx, N3, Nx, Nsa, Nx, and Mx, as well as unknown survival times and multiple primary tumors. GSRC with pathological type code 8490/3 were screened and randomly divided into training and internal validation sets in a 7:3 ratio. Information on postoperative patients with GSRC was collected from the Affiliated Tumor Hospital of Harbin Medical University (from January 1, 2002, to April 31, 2014) for external validation. The inclusion criterion was postoperative GC with a confirmed pathological diagnosis. The exclusion criteria were as follows: (1) multiple primary tumors and (2) incomplete clinical pathological information. The endpoint of the study was overall survival (OS), defined as the period from pathological confirmation of GSRC to confirmed death or the last follow-up via phone or text message.

The obtained patient clinical data included nine clinical features: age, sex, primary disease, 7th edition AJCC Stage Group (Stage), T, N, M, regional nodes optimized (RNE), and lymph node metastasis rate (LNMR). The patient stage is classified according to the 7th edition AJCC TNM staging standard. The optimal cutoff values for age and LNMR were determined using X-tile software (version 3.6.1, Yale University School of Medicine, USA) [18].

### Construction and validation of nomogram

Univariate and multivariate Cox regression models analyzed the relationship between clinical features and OS through the R language “survival” package in the training set. Nine clinical features were preliminarily screened through univariate Cox regression analysis. Samples with *P* < 0.05 were included in multivariate Cox regression analysis, and a forest map was drawn using the "ggplot2" software package. Based on the independent prognostic risk factors obtained through multivariate Cox regression analysis, the 1-, 3-, and 5-year nomograms were constructed using the “ms” package. Apply the following methods to evaluate the performance of the nomogram in the training set, internal validation set, and external validation set. The “imeROC” package was used for receiver operating characteristic curve (ROC) analysis to evaluate the sensitivity and specificity of the nomogram, and the area under the curve (AUC) was determined. The “ggplot2” package was used for visualization. The C-index and calibration curve were calculated by the “rms” package, and the decision curve (DCA) analysis was performed by the “stdca” package [19].

### Survival subgroup analysis of nomogram

A nomogram prognostic risk score (risk score) was calculated. Using the median risk score as the cutoff point, patients in the training set were divided into high- and low-risk groups. A survival analysis was conducted for each subgroup in the training set to evaluate the survival differences between the groups and the performance of the nomogram in each subgroup.

### Nomogram assisted postoperative adjuvant chemotherapy decision-making analysis

Calculate the external validation set risk score. Using the median risk score as the cutoff point, patients in the external validation set are divided into high- and low-risk groups. Perform OS analysis on patients with and without chemotherapy in the high- and low-risk groups to evaluate the performance of the nomogram in postoperative adjuvant chemotherapy decision-making.

### Statistical analysis

If the variable was of a numerical type and did not meet the normal distribution, the Kruskal–Wallis method was selected as the comparison method for the three groups. If the variable was of classification type, when the data met the theoretical frequency > 5 criterion and the total sample size was ≥ 40, the Chisq test method was used for inter-group comparison. When the data met the criteria of 1 ≤ theoretical frequency < 5 and the total sample size was ≥ 40, Yates’ correction method was used for inter-group comparison. The Kaplan–Meier method was used for survival analysis, and the log-rank method was used for inter-group difference analysis. All statistical analyses were conducted using R software (version 4.2.1). Statistical significance was set at *P* < 0.05.

### Ethical statement

The data in the SEER database is open and shared, without the need for informed consent from patients. The external validation set data has been approved by the Affiliated Tumor Hospital of Harbin Medical University.

## Results

### Data acquisition and determination of clinical features

**Fig. 1:**
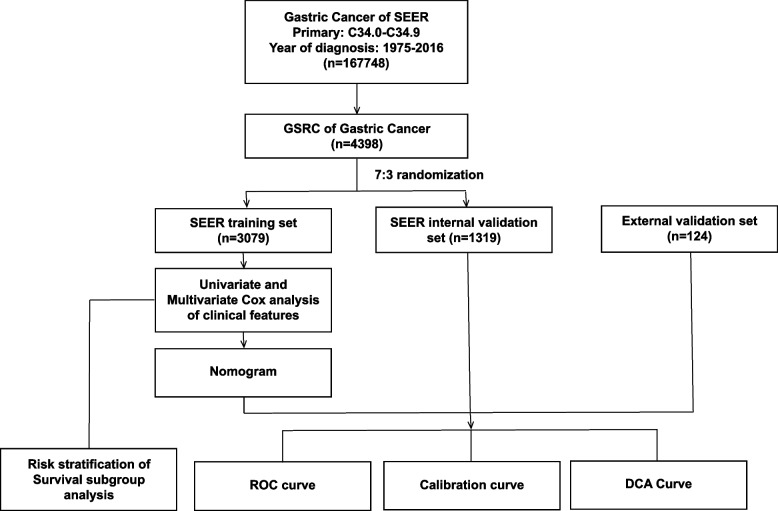
Workflow Diagram

We obtained 167,748 clinical records of postoperative patients with GC from the SEER database, and data of tumor patients with uncertain or incomplete information and multiple primary sites were removed. The study included 4,398 GSRC postoperative patients with complete information. They were randomly divided into training and internal validation sets in a 7:3 ratio, comprising 3,079 training set samples and 1,319 internal validation set samples. A total of 124 postoperative patients with GSRC were recruited from the Cancer Hospital affiliated with Harbin Medical University, including 77 males and 47 females, with a sex ratio of 1.6:1 and a median age of 52 years (24–81 years). The baseline data are presented in (Table 1), and a workflow diagram is shown in (Fig. 1).

**Table 1:** Clinical features baseline data

	SEER training set	SEER internal validation set	External validation set	*P* value	Method
	n (%)	n (%)	n (%)		
Total	3079 (68.1%)	1319 (29.2%)	124 (2.7%)		
Fustat				** < 0.001**	Chisq test
Death	905 (20%)	442 (9.8%)	66 (1.5%)		
Alive	2174 (48.1%)	877 (19.4%)	58 (1.3%)		
Futime, median (IQR)	720 (270, 1620)	630 (240, 1560)	1650 (690, 2377.5)	** < 0.001**	Kruskal–Wallis
Age				** < 0.001**	Chisq test
≥ 70	1032 (22.8%)	427 (9.4%)	5 (0.1%)		
< 70	2047 (45.3%)	892 (19.7%)	119 (2.6%)		
Gender				**0.010**	Chisq test
Female	1509 (33.4%)	601 (13.3%)	47 (1%)		
Male	1570 (34.7%)	718 (15.9%)	77 (1.7%)		
Primary				** < 0.001**	Yates' correction
Cardia	443 (9.8%)	206 (4.6%)	5 (0.1%)		
Fundus of stomach	74 (1.6%)	39 (0.9%)	2 (0%)		
Body of stomach	324 (7.2%)	139 (3.1%)	18 (0.4%)		
Gastric antrum	865 (19.1%)	361 (8%)	65 (1.4%)		
Pylorus	139 (3.1%)	52 (1.1%)	1 (0%)		
Lesser curvature of stomach	366 (8.1%)	191 (4.2%)	8 (0.2%)		
Greater curvature of stomach	178 (3.9%)	77 (1.7%)	4 (0.1%)		
Overlapping lesion of stomach	357 (7.9%)	130 (2.9%)	6 (0.1%)		
Stomach-NOS	333 (7.4%)	124 (2.7%)	15 (0.3%)		
Stage				** < 0.001**	Chisq test
Stage I	874 (19.3%)	333 (7.4%)	30 (0.7%)		
Stage II	591 (13.1%)	268 (5.9%)	28 (0.6%)		
Stage III	785 (17.4%)	318 (7%)	46 (1%)		
Stage IV	829 (18.3%)	400 (8.8%)	20 (0.4%)		
T				**0.019**	Chisq test
T1	572 (12.6%)	208 (4.6%)	17 (0.4%)		
T2	1253 (27.7%)	530 (11.7%)	56 (1.2%)		
T3	897 (19.8%)	416 (9.2%)	45 (1%)		
T4	357 (7.9%)	165 (3.6%)	6 (0.1%)		
N				0.308	Chisq test
0	953 (21.1%)	386 (8.5%)	33 (0.7%)		
1	1094 (24.2%)	483 (10.7%)	43 (1%)		
2	704 (15.6%)	282 (6.2%)	32 (0.7%)		
3	328 (7.3%)	168 (3.7%)	16 (0.4%)		
M				** < 0.001**	Chisq test
M0	2645 (58.5%)	1110 (24.5%)	122 (2.7%)		
M1	434 (9.6%)	209 (4.6%)	2 (0%)		
RNE				**0.006**	Chisq test
< 15	1436 (31.8%)	596 (13.2%)	40 (0.9%)		
≥ 15	1643 (36.3%)	723 (16%)	84 (1.9%)		
LNMR				** < 0.001**	Chisq test
0	1006 (22.2%)	401 (8.9%)	34 (0.8%)		
< 30%	683 (15.1%)	310 (6.9%)	45 (1.0%)		
≥ 30% and < 70%	679 (15%)	301 (6.7%)	22 (0.5%)		
≥ 70%	711 (15.7%)	307 (6.8%)	23 (0.5%)		
Chemotherapy				N/A	N/A
Chemotherapy	N/A	N/A	74 (59.7%)		
No chemotherapy	N/A	N/A	50 (40.3%)		

Abbreviations: *SEER* Surveillance, Epidemiology, and End Results, *AJCC* American Joint Co mmittee on Cancer, *RNE* Regional nodes examined, *LNMR* Lymph node metastasis rate

*P* < 0.05 is considered statistically significant and is represented in bold font

**Fig. 2:**
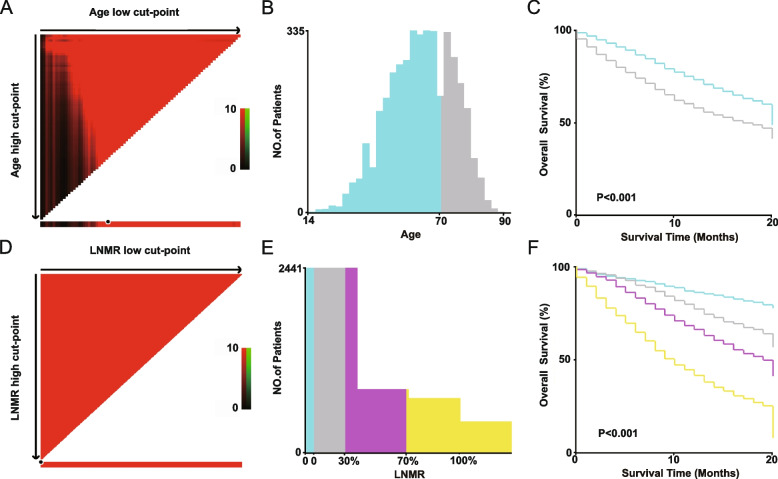
X-tile analysis determines the cutoff values for age and LNMR. **A**-**C** When the age cutoff value is 70 and (D-F) the LNMR cutoff value is 0, 30%, and 70%, respectively, it can better distinguish the OS rates of each group. Histograms and subgroup survival curve analysis were developed based on these truncated values. *P* < 0.05 is considered statistically significant

The optimal cutoff values were determined using X-tile software when the age was (≥ 70 years old, < 70 years old) (Fig. 2A–C), and the LNMR was (0, < 30%, ≥ 30%, and < 70%, ≥ 70%) (Fig. 2D-F).

### Univariate and multivariate analysis of overall survival rate in the training set

**Fig. 3:**
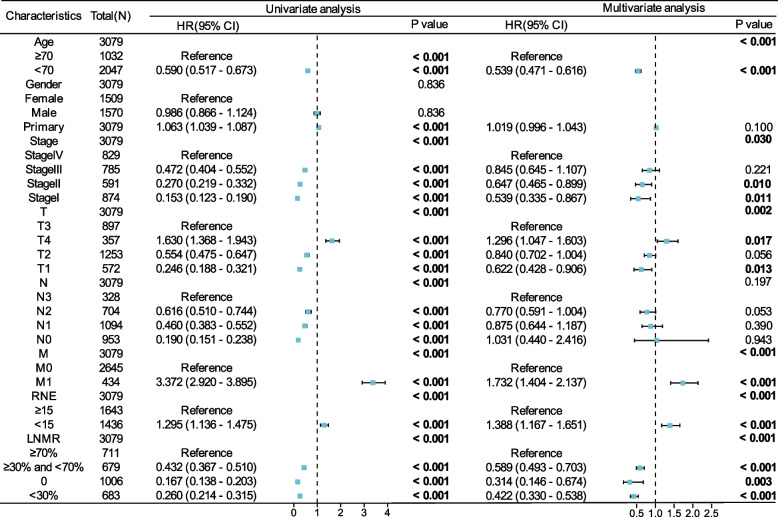
Univariate and multivariate analysis of clinical features and overall survival of GSRC postoperative patients in the training set. Abbreviation: GSRC: Gas Sign Ring Cell Carcinoma, RNE: Regional nodes optimized, LNMR: Lymph node metastasis rate, HR: Risk ratio, CI: Confidence interval. *P* < 0.05 is considered statistically significant and is represented in bold font

Univariate Cox regression analysis was conducted on nine clinical features of patients in the training set. Samples with *P* < 0.05 in the univariate Cox regression analysis were included in the multivariate Cox regression analyses to construct a risk proportional regression model (Fig. 3). Univariate analysis indicated that age, primary tumor stage, T, N, M, RNE, and LNMR were high-risk factors that affected prognosis. The results of the multivariate analysis indicated that age, stage, T, M, RNE, and LNMR were independent risk factors affecting prognosis.

### Construction and validation of nomogram

**Fig. 4:**
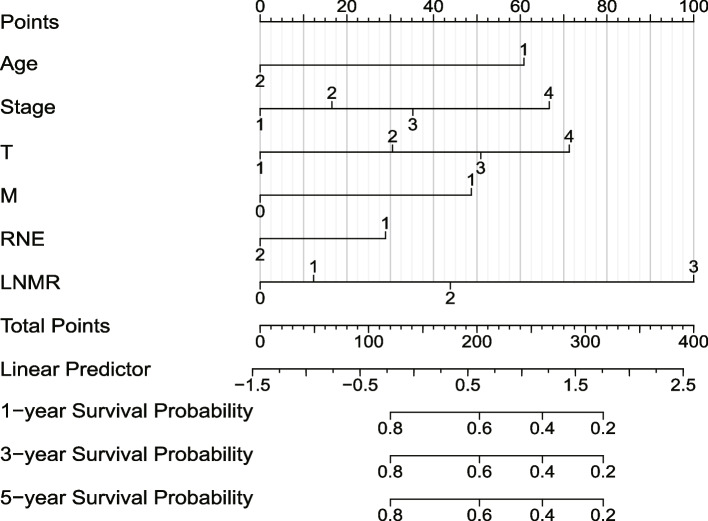
Construction of GSRC Nomogram using training set data. Points represent the individual scores corresponding to each predictive variable at different values. Features represents the values and corresponding scores of each variable in the model, Total Points represents the total score of the individual scores corresponding to all variable values, and Linear Predictor represents the linear predictive value

Based on the multivariate Cox regression analysis results, we integrated six independent risk factors (age, stage, T, M, RNE, and LNMR) using scaled line segments to construct a nomogram (Fig. 4). We added the corresponding individual scores of all variables to obtain the total score and determine the 1-, 3-, and 5-year survival probabilities corresponding to the total score. The relationship between predictive scores and OS in the training, internal validation, and external validation sets was evaluated. The results indicated that the C-index in the training, internal validation, and external validation sets was 0.741 (0.733–0.750), 0.741 (0.729–0.752), and 0.786 (0.755–0.816), respectively. The consistency of the model was good, and it could better predict the OS of patients after GSRC surgery.

**Fig. 5:**
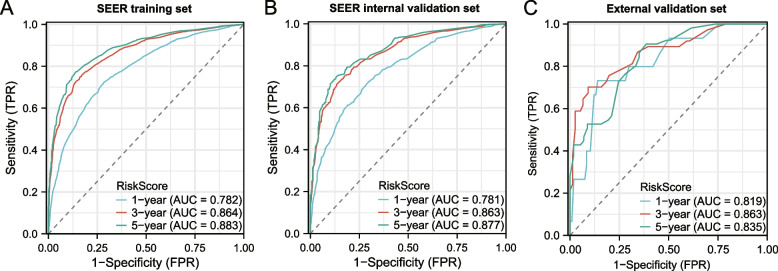
Nomogram ROC curve. **A**-**C** Nomogram ROC curves for 1, 3, and 5 years of OS in training, testing, and external validation sets. The horizontal axis represents the false positive rate (FPR) (1-specificity), and the vertical axis represents the true positive rate (TPR) (sensitivity)

**Fig. 6:**
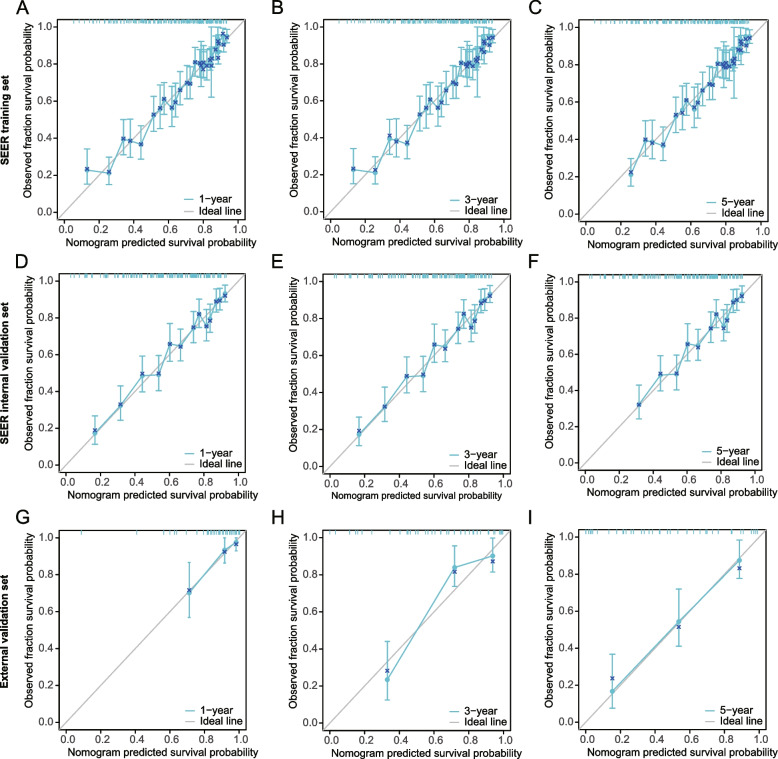
Nomogram calibration curve. **A**-**C** Nomogram calibration curves for 1, 3, and 5 years of OS in training, testing, and external validation sets. The horizontal axis represents the predicted survival probability of the model, and the vertical axis represents the actual observed survival probability. Each line represents the survival situation at each time point compared with the actual situation, and the most ideal line (diagonal line: gray). The vertical line corresponding to each line's point represents that position's confidence interval. The blue cross on each line represents the result of each point after hierarchical Kaplan Meier correction

In addition, we generated ROC and calibration curves to evaluate the efficiency and calibration ability of the nomograms in the three queues. The ROC curve results indicated that the AUC of the training sets for 1, 3, and 5 years was 0.782, 0.864, and 0.883, respectively. The AUC for the internal validation sets was 0.781, 0.863, and 0.877, and that for the external validation sets was 0.819, 0.863, and 0.835, respectively (Fig. 5A–C). The calibration curve results indicated that the predicted 1-, 3-, and 5-year OS rates in the three cohorts were highly consistent with the actual OS (Fig. 6A–I). The above results suggest that the model has good predictive performance for prognosis.

**Fig. 7:**
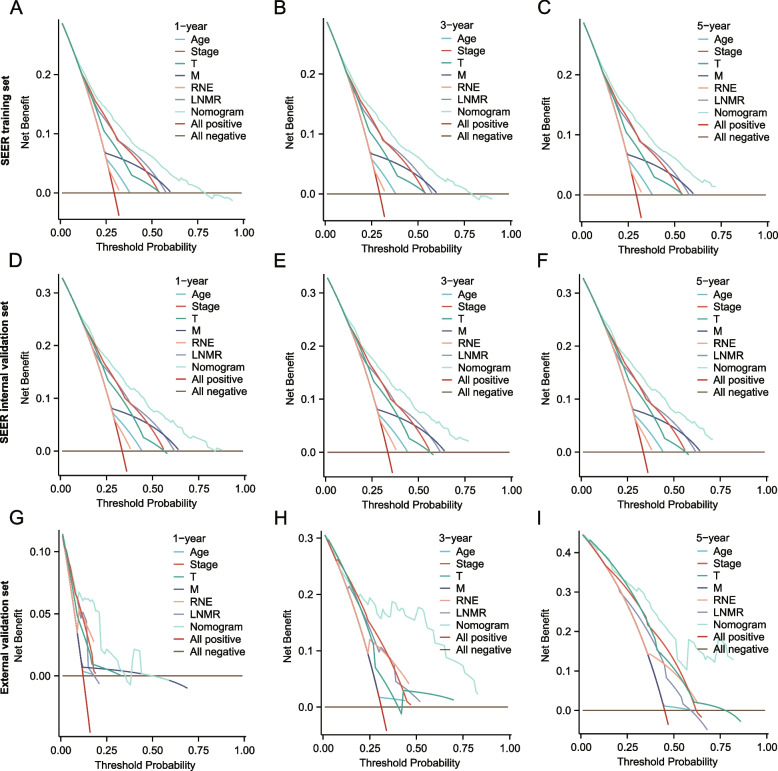
Nomogram DCA curve. **A**-**C** Nomogram DCA curves for 1, 3, and 5 years of OS in training, testing, and external validation sets. The x-axis represents the Threshold Probability, and the y-axis represents the net return

Considering that ROC and calibration curves can only evaluate the accuracy, specificity, and consistency of model predictions, their clinical utility cannot be evaluated. Therefore, we used DCA curves to integrate the preferences of patients and decision-makers into the analysis and evaluated their net clinical benefits based on the actual needs of clinical decision-making. The results indicated that using the nomogram constructed in this study to predict OS in the three cohorts would have a greater net benefit than using a single clinical feature (Fig. 7A–I).

### Survival subgroup analysis of nomogram

**Fig. 8:**
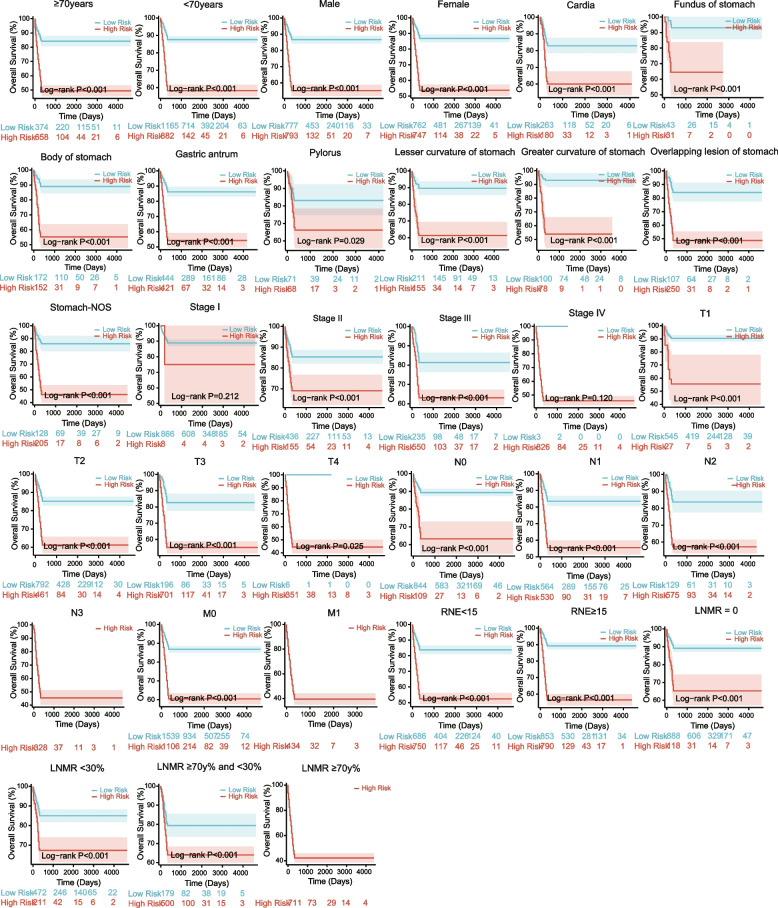
Survival subgroup analysis of Nomogram. The High-risk group represents high-risk populations, while the Low-risk group represents low-risk populations. *P* < 0.05 is considered statistically significant

The nomogram prognostic risk score was then calculated using the median riskscore as the cutoff point. Riskscore = -3.156 + Age < 70 years × (-0.618) + Primary × (0.0192) + Stage III × (-0.169) + Stage II × (-0.436) + Stage I × (-0.618) + T4 × (0.259) + T2 × (-0.175) + T1 × (-0.474) + N2 × (-0.261) + N1 × (-0.134) + N0 × (0.031) + M1 × (0.549) + RNE < 15 × (0.328) + LNMR ≥ 30% and < 70% × (-0.530) + LNMR0 × (-1.158) + LNMR < 30% × (-0.864). Patients in the training set were divided into high- and low-risk groups, and survival analysis was conducted for each subgroup in the training set. The results indicated that patients in the three subgroups of N3, M1, and LNMR ≥ 70% were all high-risk groups, and the *P* values of the Stage I and Stage IV subgroups were not statistically significant. Among the other subgroups, the risk score can better classify them into high-risk and low-risk groups (Fig. 8).

### Nomogram assisted postoperative adjuvant chemotherapy decision-making analysis

**Fig. 9:**
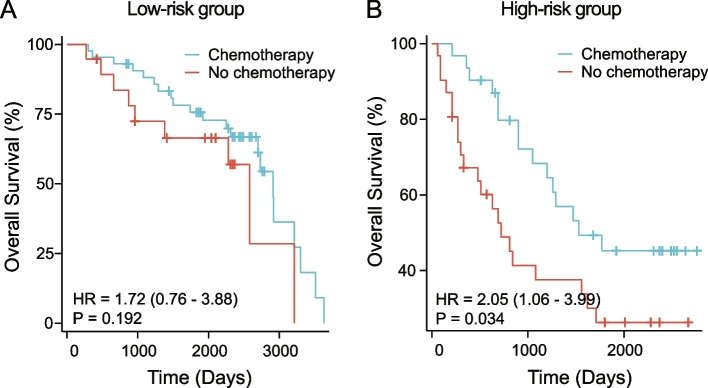
Nomogram assisted postoperative adjuvant chemotherapy decision-making analysis. The blue line represents patients who have undergone adjuvant chemotherapy after surgery. The red line represents patients who have not undergone adjuvant chemotherapy after surgery. *P* < 0.05 is considered statistically significant

Similarly, patients in the external validation set were divided into high- and low-risk groups, and OS analysis was performed on patients with and without chemotherapy in the high- and low-risk groups. The results showed that the overall survival rate of patients in the high-risk group who received chemotherapy was significantly better than those who didn’t receive chemotherapy (*P* = 0.034), while the overall survival rate of patients in the low-risk group who received chemotherapy was not significantly different from those who didn’t receive chemotherapy (*P* = 0.192). This indicates that adjuvant chemotherapy can significantly improve OS in high-risk group, while the presence or absence of adjuvant chemotherapy in low-risk group has no significant impact on OS (Fig. 9).

## Discussion

This study constructed a reliable GSRC postoperative prognosis evaluation system to make personalized prognostic judgments and clinical treatment decisions for postoperative GSRC patients.

We have combined the strengths and limitations of clinical features. First, the 8th edition of the NCCN guidelines for TNM staging requires the number of lymph nodes undergoing a pathological examination to be ≥ 15. If it is < 15, it may cause inaccurate staging because of insufficient lymph node dissection. However, owing to differences in surgical methods, anatomical structures, or complex emergency surgeries (perforation, bleeding, and obstruction), dissection is difficult, and sufficient lymph nodes cannot be obtained. Therefore, this study included the clinical features of RNE. Second, this study included the clinical features of LNMR, first proposed by Japanese scholars in 1990, which were defined as the number of lymph node metastases/number of lymph nodes sent for examination [20]. LNMR has specific advantages over N staging, such as having 15 lymph node metastases in patients with GC and 15 and 30 lymph node clearances. In most cases, a difference in prognosis exists between the two; however, the N staging is the same, which cannot accurately distinguish the prognosis of patients. However, LNMR values of 100% and 50% may better differentiate prognoses. Previous studies have shown that LNMR has a significant advantage over N staging in predicting the prognosis of GC [21, 22]. Moreover, considering that the developed system may be more applicable to evaluating patients after their first diagnosis, we did not include treatment information to guide the selection of treatment plans. Additionally, the external validation set used in this study included only single-center data from Asian populations that did not match the features of the SEER database. Therefore, race was not included as a variable in this study.

GSRC has the features of low differentiation, strong invasiveness, and poor prognosis and is often diagnosed in the middle to late stages with lymph node metastasis [11]. Some studies have analyzed the prognostic factors of patients with GSRC; however, the conclusions drawn must be confirmed. Nakamura et al. suggested that sex, age, tumor location, size, macroscopic type, and TNM stage are independent risk factors for GSRC prognosis [23]. A meta-analysis by Guo et al. [24] demonstrated that age, lymphatic invasion, and TNM stage were independent risk factors for GSRC prognosis. Additionally, meta-analyses by both Zhao et al. [25] and Zhao et al. [26] indicated that the prognosis of early GSRC patients was better than that of patients with other types of GC, whereas the prognosis of advanced GSRC patients was relatively poor. Therefore, further research is required to determine the factors that influence the prognosis of patients with GSRC.

Prognostic models are currently being developed for patients with GSRC. Chen et al. developed a highly effective nomogram prognostic evaluation system using GSRC clinical data from the SEER database. The results indicated that age, race, tumor size, T, N, and M staging, surgery, and radiation use were independent risk factors for prognosis. The nomogram prognostic evaluation system containing the above features is relatively more effective than the TNM staging system [27]; Guo et al. obtained similar results. However, both studies only conducted internal dataset validation and did not adequately validate the real-world data. Therefore, this study modified the clinical features and constructed a reliable GSRC postoperative prognosis evaluation system to guide treatment after the initial diagnosis.

Although differences occurred in the clinical features included compared with those in previous studies, the results of the nomogram prognostic evaluation system constructed in this study remained satisfactory. The consistency between the C-index and calibration curve results was still good, and the ROC curve performance at 1, 3, and 5-years was excellent. The DCA curve results indicated that using this nomogram to predict OS would increase the net benefits more than using a single clinical feature, indicating good clinical practicality. Additionally, this study conducted internal and external dataset validation, resulting in higher reliability. In the nomogram survival subgroup analysis, the prognostic evaluation system effectively distinguished most patients in the subgroups into high- and low-risk groups. The P values in Stage I and Stage IV subgroups were not statistically significant, possibly because of the small sample size of the high- and low-risk groups in Stage I and Stage IV. Patients in the three subgroups of N3, M1, and LNMR ≥ 70% are all high-risk groups, which is consistent with the real-world situation. It is worth noting that although there is no matching treatment information in the SEER database, we have followed up with matching treatment information in the external validation set. Therefore, we used this nomogram to conduct postoperative adjuvant chemotherapy decision analysis on external datasets, and the results showed that postoperative chemotherapy did not have a significant impact on OS in low-risk groups, while postoperative chemotherapy in high-risk groups significantly improved OS, thereby enhancing clinical benefits. This indicates that the nomogram may serve as a supplement to assist the TNM staging system in making decisions.

However, this study had some limitations. For example, owing to the incomplete matching of features between the SEER database and the external validation set data, we did not include information such as region, race, surgical method, immunohistochemistry, or molecular markers. In addition, the small sample size of the external validation set led to uneven patient distribution in each subgroup. In future studies, we plan to expand the sample size to further validate the model.

## Conclusions

In summary, we developed a postoperative GSRC patient nomogram prognostic evaluation system with good performance, strong practicality, and high reliability. This will help clinicians make personalized prognosis judgments and clinical treatment decisions for patients after their first diagnosis, improve their quality of life, and prolong their OS.

## Data Availability

The data from the Cancer Hospital affiliated with Harbin Medical University is not publicly available. The SEER data analyzed in this study is available at https://seer.Cancer.gov/.
